# Prolonged vasopressors therapy is associated with glucose variability after cardiac surgery

**DOI:** 10.1186/2197-425X-3-S1-A738

**Published:** 2015-10-01

**Authors:** M Svetikienė, D Ringaitiene, V Isajevas, D Gineityte, V Vicka

**Affiliations:** Vilnius University Hospital Santariskiu Clinics, Vilnius, Lithuania; Vilnius University, Vilnius, Lithuania

## Introduction

Stress hyperglycemia in the ICU environment is a highly regarded study subject since Lueven collaborators publications in 2001. Current research focused on variability of blood glucose levels suggests it being an independed predictor of bad clinical outcome. Despite the importance of the subject the genesis of the variability of the glycemia is not fully understood.

## Objectives

We aim to associate prolonged vasopressors therapy with glucose variability after cardiac surgery.

## Methods

The study was conducted in a tertiary hospital center and included patients undergoing cardiac surgery. Randomization was applied using demographic and operative criteria. First postoperative day data was used to calculate the glucose variability adopting three hypothetical markers: difference between highest and lowest blood glucose level (GluDiff), SD of mean glucose (GluSD) and a group of patients having both hyper and hypoglycemia (GluHH). Prolonged vasopressors therapy (PVT) was defined as a use of sympatomimetic drugs more than 24 hours postoperatively. Estimation coefficients (B) of PVT were calculated using decimiles for GluDiff and GluSD. Appropriate statistical tests were used to adjust for the factors affecting glucose concentration.

## Results

Study included 779 patients, 521 of them in the randomized group. PVT was diagnosed in 201 (38,6%) and was linked to mean glycemia (7,67 ± 1,31 vs 6,99 ± 1,17 ± p < 0,001). All three glucose variability markers were associated with PVT (p < 0,01). Analyzing the decimiles of GluDiff (B10th=-2,1 to B90th=1,92) and GluSD (B10th=-2,82 to B90th=1,8) in ordinal regression revealed growth of estimates linked to the PVT (p < 0,05). This association persisted in multivariate regression analysis adjusted for aortic clamping time, bypass time, gender, age and other predictors of glucose variability. Furthermore, rate of PVT was higher in GluHH group (32 (61,5%) vs 129 (37%) p=0,001) suggesting OR of 2,73 (CI95% 1,49-4,97 p=0,001). In multivariate regression analysis adjusted for aforementioned criteria PVT persisted as an independent predictor of GluHH (OR = 2,38 CI95% 1,18-4,8 p=0,016).

## Conclusions

We report the association between prolonged vasopressors therapy and increased blood glucose level. Furthermore, we establish the link between glucose variability and use of sympatomimetics. However our study lacks the exact doses of the drugs used so we note the need of further research in this field.

